# The efficacy of human placenta-derived mesenchymal stem cells on radiation enteropathy along with proteomic biomarkers predicting a favorable response

**DOI:** 10.1186/s13287-017-0559-5

**Published:** 2017-05-02

**Authors:** Young-Min Han, Jong-Min Park, Yong Soo Choi, Hee Jin, Yun-Sil Lee, Na-Young Han, Hookeun Lee, Ki Baik Hahm

**Affiliations:** 1 CHA Cancer Prevention Research Center, CHA University, CHA Bio Complex, 335 Pangyo-ro, Bundang-ku, Seongnam, Kyunggi-do 463-712 South Korea; 2 Department of Applied Bioscience, CHA University, Seongnam, South Korea; 3 Graduated School of Pharmaceutical Sciences, Ewha Womans University, Seoul, South Korea; 4 Lee Gil Ya Cancer and Diabetes Institute, College of Pharmacy, Gachon University, Incheon, South Korea; 5 Digestive Disease Center, CHA Bundang Medical Center, CHA University, Seongnam, South Korea

**Keywords:** Placenta-derived mesenchymal stem cells, Radiation enteropathy, Regeneration, Biomarkers

## Abstract

**Background:**

Radiation enteropathy is a common complication in patients with abdominopelvic cancer, but no treatment has yet been established. Stem cell therapy may be a viable therapeutic option because intestinal stem cells are highly vulnerable to ionizing radiation (IR) and stem cell loss explains its intractability to general treatment. Here, we investigated either prophylactic or therapeutic efficacy of human placenta-derived mesenchymal stem cells (hPDSCs) against radiation enteropathy and could identify biomarkers predicting a favorable response to stem cell therapy.

**Methods:**

We challenged a radiation-induced enteropathy model with hPDSCs. After sacrifice, we checked the gross anatomy of small intestine, histology gross, and analyzed that, accompanied with molecular changes implicated in this model.

**Results:**

hPDSCs significantly improved the outcome of mice induced with either radiation enteropathy or lethal radiation syndrome (*P* < 0.01). hPDSCs exerted inhibitory actions on inflammatory cytokines, the re-establishment of epithelium homeostasis was completed with increasing endogenous restorative processes as assessed with increased levels of proliferative markers in the hPDSCs group, and a significant inhibition of IR-induced apoptosis. The preservation of cells expressing lysozyme, and Musashi-1 were significantly increased in the hPDSC treatment group. Both preventive and therapeutic efficacies of hPDSCs were noted against IR-induced enteropathy. Label-free quantification was used to identify biomarkers which predict favorable responses after hPDSC treatment, and finally glutathione S-transferase-*mu* type, interleukin-10, and peroxiredoxin-2 were validated as proteomic biomarkers predicting a favorable response to hPDSCs in radiation enteropathy.

**Conclusions:**

hPDSCs may be a useful prophylactic and therapeutic cell therapy for radiation enteropathy.

**Electronic supplementary material:**

The online version of this article (doi:10.1186/s13287-017-0559-5) contains supplementary material, which is available to authorized users.

## Background

Diagnostic and therapeutic applications of irradiation (IR) are very important and useful in modern medicine [1–4]. However, IR can induce DNA damage, chromosomal aberrations, cell cycle arrest or cell death and ultimately lead to the development of malignancies [5, 6]. Though radiation therapy is commonly administered to the abdomen and pelvis of patients with gastrointestinal (GI), urological, and gynecological cancers, the toxicity of radiation to normal intestine remains the biggest obstacle. Within the gut, intestinal stem cells (SCs) and endothelial cells are especially radiosensitive, and it is common for them to be lost following IR. The loss of these cells can result in a disrupted mucosal barrier and insufficient blood supply to the gut [7, 8], and lead to troublesome radiation enteropathy. Once radiation enteropathy develops, patients may suffer from vomiting, diarrhea, abdominal pain, and bleeding [7], but unfortunately no preventive or restorative treatment modalities are available.

Since current management of radiation enteropathy is mainly supportive or surgical in those few patients that experience sustained GI bleeding [9] and there is no specific approach to prevent it, we conducted this study under the hypothesis that supplementing human placenta-derived mesenchymal stem cells (hPDSCs) can limit IR-induced enteropathy and identifying potential biomarkers to predict favorable responses to these hPDSCs can benefit clinical trials. Supported with diverse promising cell therapies including the engraftment of hematopoietic stem cells in hematological malignancies [10], the improvement of bone growth by matrix synthesis in osteogenesis imperfecta [11], the reduction of GI disorders in patients with severe resistant graft-versus-host disease (GVHD) [12], the reversion of colon peritonitis in patients with GVHD [13], and the treatment of rectovaginal and perianal fistulas in patients with Crohn’s disease [14], in the current study, we performed this proof-of-concept study to evaluate whether hPDSC administration efficiently prevents or treats radiation enteropathy through acknowledged mechanisms of restorative, anti-oxidative, anti-inflammatory, and anti-apoptotic actions of stem cells. As results, in addition to significant therapeutic effects, interleukin-10 (IL-10), Tissue inhibitor of metalloproteinases (TIMP-1), glutatione-S-transferase mu (GST *mu*), NADPH-oxidase4 (NOX-4), and peroxiredoxin-2 (PRDX-2) were drawn as biomarkers predicting favorable response to hPDSCs.

## Methods

### Cell culture

hPDSCs were provided from CHA Biotech (Seongnam, Korea). The hPDSC line was cultured in α-MEM medium containing 1 μg/ml heparin, 25 mg/ml fibroblast growth factor 4, 10% (v/v) fetal bovine serum and 100 U/ml penicillin. Cells were maintained at 37 °C in a humidified atmosphere containing 5% CO_2_.

### Animals and study protocol

**Fig. 1:**
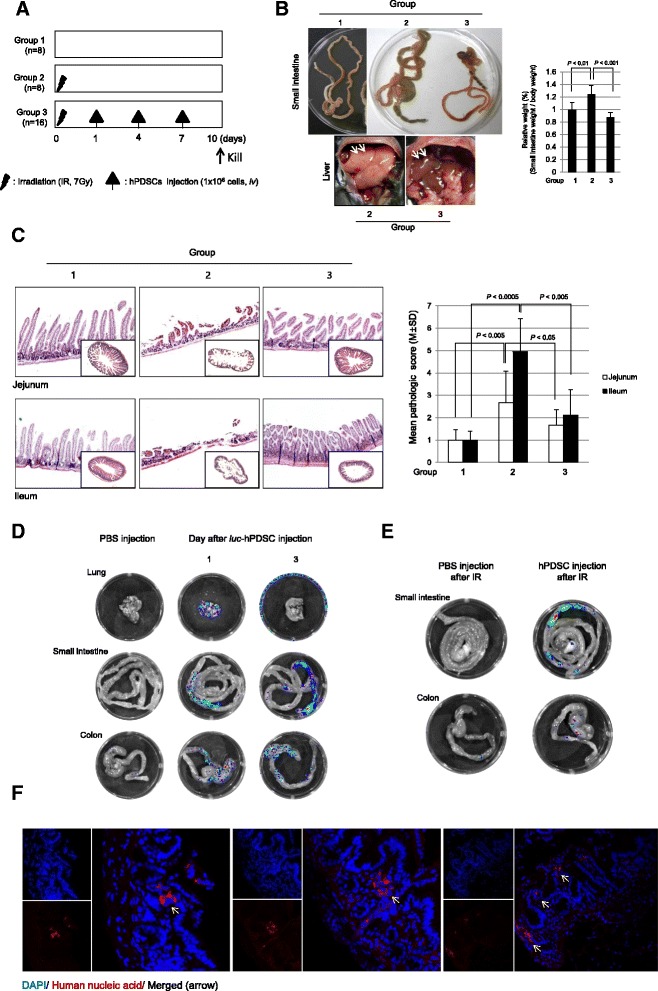
The gross, structural studies of intestine according to group and injected hPDSCs, which are localized to target tissue in irradiation-induced small intestine damage. **a** The animal experimental procedure. **b** Representational gross lesions according to group. To check the degree of swelling, we measured the weight of the small intestine in each group. **c** The pathological sections were observed and histological score was estimated by three independent blinded pathologists. Microscopic pathologies according to group: normal control (group 1), irradiation (group 2) and irradiation with hPDSCs (group 3). Results are representative pathologic images of three independent samples (×100 magnification). **d** To determine where injected hPDSCs localized in tissue through intravenous injection, we transfected the *luc*-vector to hPDSCs and then measured luciferase activity through Xenogen. Fluorescence demonstrated *luc*-positive hPDSCs treatment in lung, small intestine and colon. **e** To check the difference between injected cells in normal status and injected cells in irradiated status, we measured luciferase activity using Xenogen. **f** To confirm the localization and status for injected hPDSCs we stained tissue slides 3 days after hPDSC injection by immunofluorescence staining (IHC-F). We collected tissues 3 days after hPDSC injection of the animals. In IHC-F staining, DAPI staining is *blue* and human nucleic acid staining is *red*. The *yellow arrows* indicate injected hPDSCs (×400 magnification). Results are representative of three independent experiments. *hPDSCs* human placenta-derived mesenchymal stem cells, *IR* ionizing radiation

C57BL/6 J mice were purchase from Central Lab. Animal Inc. (Seoul, Korea). Six-week-old female C57BL/6 J mice were fed sterilized commercial pellet diets (Biogenomics, Seoul, South Korea) and sterile water ad libitum, and housed in an air-conditioned biohazard room at a temperature of 24 °C. Group 2 and group 3 mice were subjected to a lethal dose of irradiation. Mice received total body irradiation at a dose of 7 Gy using Gammacell 300 (Best Theratronics, Kanata, ON, Canada). This study was performed under the guidelines for use and care of laboratory animals and approved by the Institutional Animal Care and Use Committee (IACUC) of the Ewha Womans University (approval number: IACUC 2013-01-048). Animal survival was monitored every 12 hours. One group of ten mice (group 1 and 2) was given intravenous injections of PBS (iNtRON Biotechnology, Seoul, South Korea) three times for 10 days. Group 3 was given intravenous injections of hPDSCs (1.0 × 10^6^/200 μl) three times for 10 days (Fig. 1a). At 10 days after irradiation, following euthanasia, the small intestines were collected, opened longitudinally, washed with sterile PBS, and tissues were analyzed following various methods. Another set of animals was prepared to document the efficacy of hPDSCs against 7 Gy radiation-induced small intestinal damage (Fig. 4a). There were five animal groups (*n* = 8/each group); group I as the normal control, group II as the radiation-induced damage group, group III receiving hPDSCs three times before before 7 Gy irradiation, group IV receiving hPDSCs three times after 7 Gy irradiation, and group V receiving hPDSCs three times before irradiation and three times after irradiation. Animal survival was monitored every 12 hours. At 10 days after irradiation, following euthanasia, the small intestines were collected, opened longitudinally, washed with sterile PBS, and tissues were analyzed following various methods.

### Antibodies

Primary antibodies for Western blotting and immunohistochemistry were purchased as follows: β-actin, Lamin B, cyclooxygenase (COX-2), nitric oxide synthase (iNOS), p53, cytochrome c, Survivin, epidermal growth factor receptor (EGFR), Ki-67 and cluster of differentiation 31 (CD31) antibodies were purchased from Santa Cruz Biotechnology (Dallas, TX, USA), phosphorylated signal transducer and activator of transcription 3 (STAT3), γ-H2AX, Bax, B-cell lymphoma 2 (Bcl-2), cleaved caspase-3, cleaved caspase-8, poly (ADP-ribose) polymerase (PARP), lysozyme and Musashi-1 from Cell Signaling Technology, Inc.(Danvers, MA, USA).

### Reverse transcriptase PCR (RT-PCR), real-time PCR and Western blots

This assay was carried out as previously described ([15, 16]). The sequences of primers are listed in Additional file 1: Table S1.

### Immunohistochemical staining

After paraffin blocks were dewaxed and rehydrated with graded alcohol, tissue sections were heated in pressure jars filled with 10 mM/L citrate buffer in a microwave for 10 minutes. Slides were cooled in water for 15 minutes and washed in PBS. The slides were incubated overnight with the primary antibody. After incubation, a subsequent reaction was formed using a Vector kit (Vector Laboratories, Inc., Burlingame, CA, USA). Finally, the slides were incubated with 3, 3’-diaminobenzidine (Invitrogen Life Technologies, Carlsbad, CA, USA) and counterstained with hematoxylin (Sigma-Aldrich, St. Louis, MO, USA).

### Terminal deoxynucleotidyl transferase-mediated dUTP nick-end labeling (TUNEL) staining

Apoptosis was visualized using a terminal deoxynucleotidyl transferase (*TdT*) fRAGel DNA fragmentation detection kit (Oncogene Research Products, La Jolla, CA, USA). To determine the apoptotic index in each group, TUNEL-immunostained sections were scanned under low-power magnification (×100) to locate the apoptotic hotspots.

### Measurement of luciferase activity in vivo using Xenogen luminometer

Cultured hPDSCs were seeded at a concentration achieving 80% confluence in six-well plates for 24 hours prior to transfection. The cells were transiently transfected with 0.2 μg/well of a translucent luciferase vector. After transfection, collected cells suspended in PBS were injected into mice through intravenous injection. After 1 day we injected luciferrin as substrate for luciferase vector into mice and 5 minutes later measured the luciferase activity using an Xenogen luminometer (Perkin Elmer, Waltham, MA, USA).

### Proteomics analysis

Biomarker protein expression of tissues according to animal experimental group were determined using label-free quantification to compare the protein profiles in three individuals with normal intestinal tissue, irradiated tissue, and injected hPDSCs in irradiated tissue. A computational framework and tools for discovery-based liquid chromatography tandem-mass spectrometry proteomics was applied to draw the biomarkers.

### Statistical analysis

All the experiments in this study were repeated more than thrice and the results are expressed as the mean ± standard deviation. The data were analyzed by one-way analysis of variance, and the statistical significance between groups was determined by Duncan’s multiple range test. Statistical significance was accepted at *P < 0.05*.

## Results

### Significant therapeutic effects of hPDSCs on radiation enteropathy

Six-week-old female mice, of C57BL/6 background, were exposed to 7 Gy IR to provoke damage to the small intestine (group 2 and group 3), among which hPDSCs (1 × 10^6^/200 μl) were administered via tail vein at day 1, 4, and 7 after IR and they were sacrificed 10 days after IR (group 3) (Fig. 1a). As noted in Fig. 1b and c, small intestines were susceptible to 7 Gy IR, yielding either significantly increased wet weights of whole small intestine (*P* < 0.01, Fig. 1b) or significantly increased pathological scores (*P* < 0.0005, Fig. 1c). In detail, significant loss of small intestinal villi, intense enteritis, marked mucosal ulcerations, and even intestinal perforations were noted in jejunum or ileum after IR in group 2. However, when mice were treated with hPDSCs, 1, 4, and 7 days after IR, there were either significant decreases in wet weights of small intestine (*P* < 0.001) or decreases in pathological scores assessed in jejunum or ileum, separately (*P* < 0.005, Fig. 1c). Blood counts were taken and IR led to significant leukopenia and anemia (*P* < 0.0005, data not shown), feasibly due to bone marrow suppression, and intestinal bleeding. On gross examination of liver surface color by group, a very pale color was noted in group 2 compared with group 3 (Fig. 1b, *arrows*), signifying that hPDSC administration maintained normal liver surface color in addition to the unchanged wet weights of small intestine compared to the control IR group (*P* < 0.001). Based on pathological score system (Additional file 1: Table S2), loss of intestinal villi accompanied with inflammatory cell infiltrates was noted in jejunum and ileum of group 2 (*P* < 0.005), whereas the intestinal villi structure and mild degree of enteritis in jejunum and ileum were maintained in group 3 despite IR treatment (*P* < 0.005) (Fig. 1c). These significant differences in either gross or pathological scores between group 2 and group 3 lead us to investigate whether injected hPDSCs migrated to site of damage to exert these significant rescuing effects against IR damage and, if so, the length of time these infused cells survived within the intestine. In order to track administered stem cells, hPDSCs were transfected with *luc*-DNA vector and collected after 24 hours. After incubation for approximately 5–10 minutes to allow for circulation throughout the body, localization was then documented following injection of luciferrin (luciferase substrate). Mice were then sacrificed and the lungs, small intestine, and colon were collected, placed on a plate, and moved to Xenogen as the bioluminescence imaging system to measure luciferase activity in each organ tested (*n* = 5). As seen in Fig. 1d, 1 day after hPDSC administration, luciferase expression was highly noted in the lung, small intestine, and colon (Fig. 1d). Three days later, luciferase activities were only observed in the small intestine and colon and all luciferase activity disappeared after 3 days. Next, Luc-DNA vector-transfected hPDSCs were administered to IR-treated mice (*n* = 5) to compare the non-irradiated and irradiated condition. There was a difference in luciferase activities according to irradiation condition, that is, a significantly increased presence of hPDSCs was noted in the small intestine of IR-treated mice compared to that of control mice (Fig. 1e), suggesting that hPDSCs homed in significantly on the intestine following IR administration and stayed for up to 3 days. Since hPDSCs originate from humans, we were able to validate the in situ existence of hPDSCs in the small intestine by performing confocal observation of tissue stained with human nucleic acids using immunohistochemistry. As seen in Fig. 1f, human nucleic acids (*red spots*) were present along with mouse nucleic acids (*blue spots*), and located in radiation-damaged villi and the nascent inflamed area of lamina propria (Fig. 1f).

### Anti-inflammatory actions of hPDSCs explain the rescuing action against radiation enteropathy

**Fig. 2:**
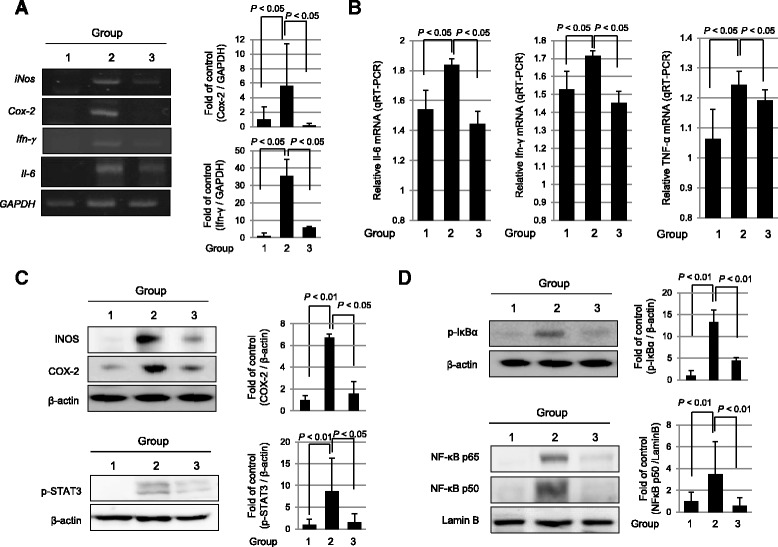
hPDSCs inhibit the expression of inflammation-associated factors in irradiated intestine. **a** RT-PCR for the proof of inflammatory cytokines in the radiation injury. Data showed RT-PCR results for checking the expression of iNOS, COX-2 and IFNγ. **b** The expression of inflammatory markers IL6, IFNγ and TNF-α were measured by real-time PCR. **c** The expression of iNOS, COX2, phosphorylated STAT3 and β-actin (as loading control) in protein extracts of each according group measured with Western blotting. **d** Nuclear extracts were performed by immunoblotting with antibodies of p65, p50, and lamin B. Cytoplasm extracts were anti-phosphorylated IκBα and β-actin. Results are representative of three independent samples. Western blotting analysis was performed with indicated antibodies. All *bars* represent the mean and SD of triplicate values respectively. *COX-2* cyclooxygenase-2, *IFN-γ* interferon gamma, *IκBα* inhibitor of kappa B, *IL-6* interleukin 6, *iNOS* nitric oxide synthase, *IR* ionizing radiation, *NF-κB* nuclear factor-κB, *p-STAT3* phosphorylated signal transducer and activator of transcription 3, *TNF-α* tumor necrosis factor alpha

We measured the changes of iNOS, COX-2, interferon gamma (IFN-γ), and interleukin (IL)-6 and compared their expression among the groups. As seen in Fig. 2a, the mRNA expressions of *iNos*, *COX*-2, *IFN*-*γ*, and *IL*-6 were significantly increased following IR, whereas their expressions were significantly decreased in group 3 administered with hPDSCs (*P* < 0.05, Fig. 2a). The decrease of IL-6, IFN-γ, and tumor necrosis factor alpha (TNF-α) mRNA levels following hPDSC administration was then confirmed by real time-PCR (*P* < 0.05, Fig. 2b). Western blots for iNOS, COX-2, and phosphorylated STAT3 were also conducted and compared across groups (Fig. 2c). As results, expression levels of COX-2 and iNOS were significantly increased in group 2, but significantly decreased in group 3 (*P* < 0.05). Phosphorylated STAT3 was also significantly increased in group 2, but significantly decreased following hPDSC administration (*P* < 0.05). Changes in redox-sensitive transcription factor and nuclear factor kappa B (NF-κB) expressions were significantly increased in group 2 (*P* < 0.01, Fig. 2d) accompanied with significantly increased levels of phosphorylated inhibitor of kappa B (IκBα) in cytoplasmic extracts from group 2 (*P* < 0.01). These findings were further validated with significantly increased expressions of NF-κB p50 and p65 in nuclear fractions. However, these activations of NF-κB were significantly decreased in group 3 (*P* < 0.01, Fig. 2d).

### Restorative actions of hPDSCs led to significant therapeutic outcome against radiation enteropathy

**Fig. 3:**
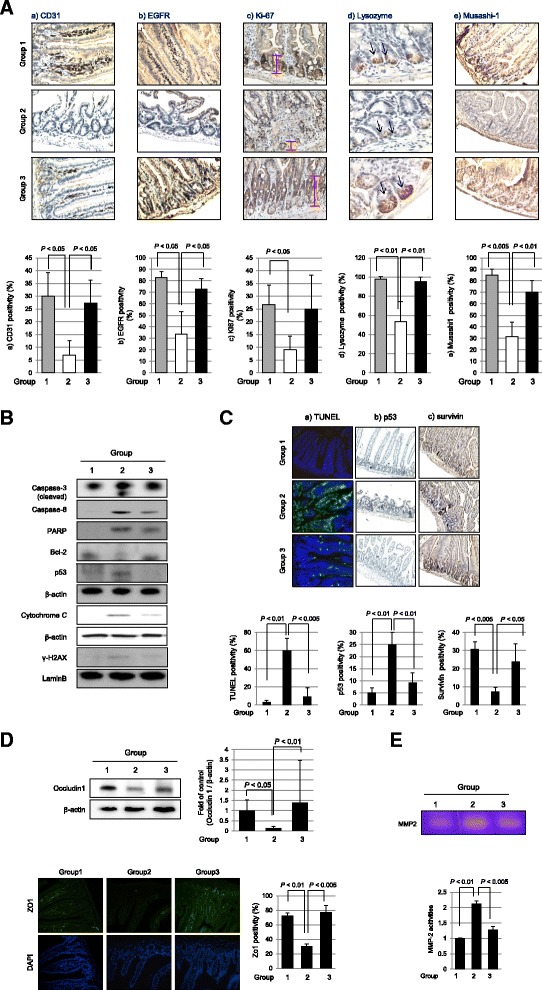
hPDSCs promote tissue regeneration and restore original stem cells. **a** Animal tissue slides were stained with CD31, EGFR and Ki67 to detect proliferation activity and to confirm stem cell existence, we checked the expression of lysozyme, as a Paneth cell marker, and Musashi-1, as an intestinal stem cell marker, which were measured by immunohistochemistry (×400 magnification). **b** Western blotting analysis was performed with indicated antibodies. Mitochondrial extracts were immunoblotted with anti-cytochrome c and β-actin. All *bars* represent the mean and SD of triplicate values respectively. **c** The TUNEL assay, p53, and survivin staining to confirm the apoptosis or anti-apoptosis, respectively (×400 magnification). All *bars* represent the mean and ST of triplicate values respectively. **d** *Upper data* showed that Occuludin1 as adhesion molecule expression using Western blotting. In *upper data*, animal tissue slides stained with ZO-1 antibody to determine adhesion index (magnification at × 400). Cell nucleus was stained with DAPI. All *bars* represent the mean and SD of triplicate values respectively. **e** Zymography for MMP-2 activity. Gelatinolytic activity was measured by zymography using small intestine homogenate. All these data are representative of three independent experiments. *Bcl-2* B-cell lymphoma 2, *CD31* cluster of differentiation 31, *EGFR* epidermal growth factor receptor, *MMP-2* matrix metalloproteinase-2, *PARP* poly (ADP-ribose) polymerase, *TUNEL* terminal deoxynucleotidyl transferase-mediated dUTP nick-end labeling

Since proliferative and regenerative actions are basic features of stem cells, we selected five targets explaining the significant rescuing action of hPDSCs against radiation enteropathy, CD31 (angiogenic activity [17]), EGFR and Ki-67 (regenerating action [18]), and lysozyme and Musashi-1 (Paneth cell and stemness [18, 19]). As shown in Fig. 3a, significant decrease in CD31 and EGFR expression with radiation damage were significantly restored following the administration of hPDSCs (*P* < 0.05). The heights of Ki67-positive crypts were significantly decreased in group 2 (*P* < 0.01), but their levels were significantly preserved in spite of IR, suggesting that hPDSC infusion faithfully contributed to the regeneration of the ulcerated tissue structure, reflected with Ki67-positive cells. When we performed immunostaining for stem cell markers including lysozyme and Musashi-1, all of these stemness markers were all significantly decreased in group 2 (*P* < 0.05), but restored in group 3 following the administration of hPDSCs (*P* < 0.01). In detail, the levels of lysozyme antibodies denoting Paneth cells, implicated in either defending epithelial maintenance or cell renewal [20], were significantly decreased in group 2 (*P* < 0.01), whereas their expression was significantly increased in group 3 (*P* < 0.01). Musashi-1 expression was also significantly decreased in group 2 (*P* < 0.005), but its expression was significantly increased in group 3 (*P* < 0.01, Fig. 3a). IR treatment results in oxidative stress and induced apoptosis in affected organs. Western blotting was done to compare the expression of apoptotic molecules including cleaved caspase-8 or caspase-3, PARP, cytochrome *c* release, p53, and γ-H2AX. As seen in Fig. 3b, group 2 showed increased expressions of these apoptotic executors or prone gene expression except Bcl-2, but these results were mitigated by the administration of hPDSCs. These molecular changes were further validated with TUNEL and immunohistochemical staining for p53 and survivin (Fig. 3c). Ten days after irradiation, we observed a six- and fivefold increase in apoptotic epithelial cells in the small intestine using TUNEL assay and p53 staining, respectively (*P* < 0.01, Fig. 3c). However, hPDSC infusion into the irradiated mice group decreased the percentage of apoptotic cells (*P* < 0.005). The number of survivin (as anti-apoptotic factor)-positive cells in the crypt compartment returned to values close to the normal control in the hPDSC-treated group (*P* < 0.05), while survivin-positive cells were significantly diminished in the irradiated group (*P* < 0.005, Fig. 3c). Changes in tight junction were also principally implicated in IR intestinal damage. As shown in Fig. 3d, the expressions of occludin-1 or ZO-1 were both significantly decreased in group 2 (*P* < 0.01), but these expressions were significantly maintained in group 3 *P* < 0.01). Lastly, when matrix metalloproteinase-2 (MMP-2) protease activities were measured by zymography, significantly increased activities of MMP-2 were shown in group 2 (*P* < 0.01), but significantly decreased in group 3 (*P* < 0.005, Fig. 3e).

### Prophylactic effects of hPDSCs against either radiation enteropathy or lethal radiation syndrome

**Fig. 4:**
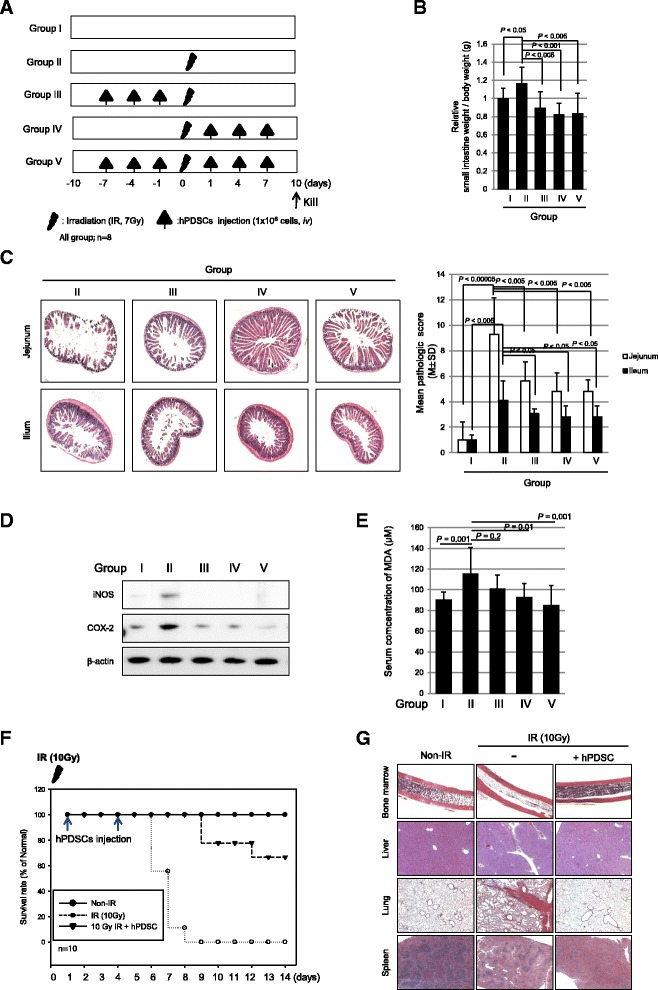
The hPDSC injection following point of time on a radiation-induced small intestinal damage model. **a** The animal experimental procedure. **b** On day 10 after irradiation, relative analysis measurements of weight of small intestine. Each value represents the average of eight independent measurements per group. **c** Histologic structures of radiation-induced intestinal damage following indicated groups. The estimated histological score by three independent blinded pathologists. Results are representative pathologic images of three independent samples (×100 magnification). **d** The expression of iNOS, COX2, and β-actin (as loading control) in protein extracts of each according to group measured with Western blotting. **e** The concentration of MDA in experimental groups. Results are presented as the mean and SD of triplicate values respectively. **f** The hPDSC injection extends life in an animal model of radiation-induced small intestine disorders: C57BL6 mice were subjected to lethal whole body irradiation at a 10 Gy dose. hPDSCs were administered by intravenous infusion. Controls received vehicle. Time points of interventions are given above the survival plots. *P* value determined by *t* test. **g** Mice, *n* = 8 in each group, were randomly sacrificed at 7 days after irradiation to obtain femur, lung, liver, and spleen for H&E staining (×400 magnification). Results are representative of eight independent samples. *COX-2* cyclooxygenase-2, *hPDSCs* human placenta-derived mesenchymal stem cells, *iNOS* nitric oxide synthase, *IR* ionizing radiation

In order to know whether hPDSCs could prevent IR-induced intestinal damage, further studies were conducted using additional groups, group III as pre-administration, group IV as co-administration, and group V as both pre- and post-administration (Fig. 4a). As results, groups III, IV, and V all showed significant rescuing outcomes from radiation-induced intestinal damage (Fig. 4b and c). Regarding the changes of wet weights of small intestine, as seen in Fig. 4b, all hPDSC-treated groups, irrespective of administration timing, showed significant protection against 7Gy IR (*P* < 0.005). These beneficial effects of hPDSCs were also verified to be statistically significant on pathological scoring (*P* < 0.005, Fig. 4c), even though results indicated a distinction of effective extent between each group. Western blots of iNOS and COX-2 consistently showed significantly attenuating effects irrespective of the administration timing of hPDSCs (Fig. 4d). Since IR causes intestinal damage through oxidative stress, serum concentration of lipid peroxidation, as measured by MDA levels, was most highly elevated in group II compared to group I (*P* < 0.001, Fig. 4e). On the other hand, group IV and V showed significantly decreased levels of serum MDA compared to group II (*P* < 0.01, Fig. 4e). Beside the intestinal protection observed following the administration of hPDSCs, we investigated whether hPDSCs can rescue mice from system radiation syndrome. For these analyses, mice (*n* = 10) were exposed to 10 Gy and mortality was tracked with irradiation alone and a group receiving 1.0 × 10^6^ hPDSCs via tail vein. One and 4 days after 10 Gy IR, as seen in Fig. 4f, IR led to about 50% mortality within 7 days and almost 100% lethality after 9 days. However hPDSCs administration after 10 Gy IR maintained 100% survival up to 9 days and 70% survival up to 15 days (*P* < 0.001, Fig 4f). Upon necroptic evaluation, 10 Gy IR provoked serious radiation syndrome manifested with bone marrow failure as evidenced by acellular marrow, extensive hepatic necrosis, respiratory failure due to extensive pneumonitis, and splenic hypoplasia (Fig. 4g). On the other hand, following administration of hPDSCs, all of these lethal changes imposed by the 10 Gy IR were significantly recovered in bone marrow, liver, lung, and spleen, respectively (Fig. 4g). Conclusively, hPDSC afforded significant protection from either radiation-induced small intestinal damage or lethal radiation syndrome.

### Label-free quantification analysis to discover significant proteomic biomarkers implicated in rescuing action of hPDSCs against radiation enteropathy

**Fig. 5:**
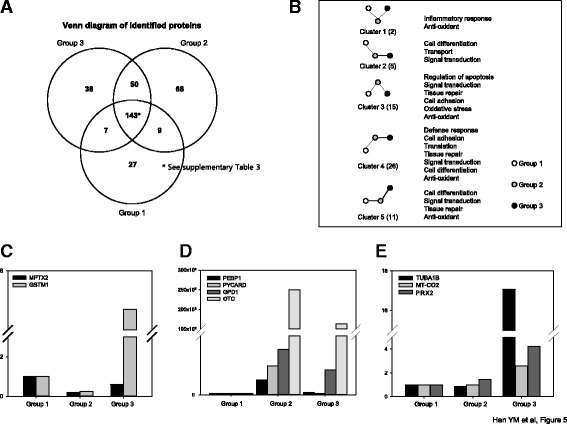
Label-free quantification analysis to detect biomarkers. **a** Venn diagram of identified proteins according to groups. **b** Gene ontology categories and the number of genes with expression differences are depicted in the *boxes*. **c**-**e** Novel biomarkers of each group following Table 1. *GPD1 g*lycerol 3-phosphate dehydrogenase [NAD (+)], cytoplasmic, GST *mu 1*, glutatione-S-transferase mu 1, *MT-CO2* cytochrome c oxidase subunit 2*OTC o*rnithine carbamoyltransferase, mitochondria *PEBP1* phosphatidylethanolamine-binding protein 1; *PRDX-2* peroxiredoxin-2, *PYCARD* apoptosis-associated speck-like protein containing CARD, *TUBA1B* tubulin-α-1B chain; *MPTX* mucosal pentraxin protein

**Fig. 6:**
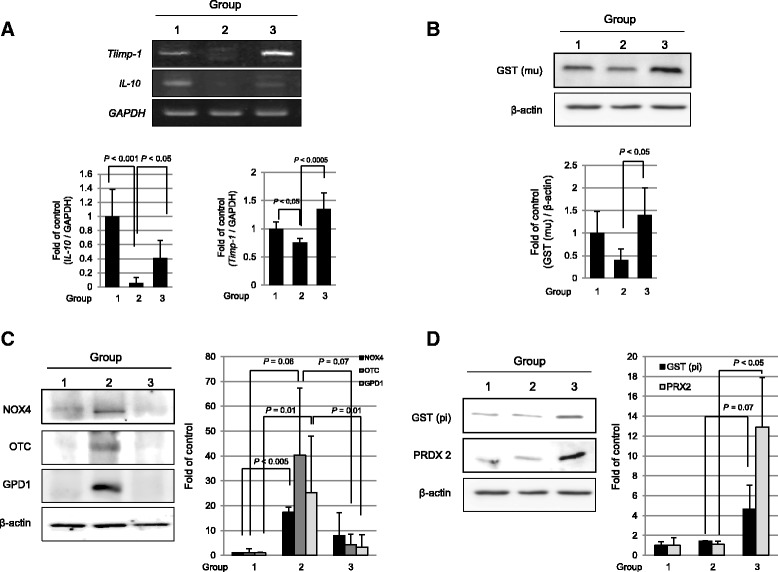
Validation of identified target from proteomics analysis. **a** The expressions of IL-10 and TIMP1 were investigated by RT-PCR. **b** The GST (*mu*) levels were determined by Western blotting on experimental groups. **c** The NOX4, OTC, and GPD levels were determined by Western blotting on experimental groups. **d** The GST (pi) and PRDX2 levels were determined by Western blotting on experimental groups. *GPD1 g*lycerol 3-phosphate dehydrogenase [NAD (+)], cytoplasmic, GST *mu 1*, glutatione-S-transferase mu 1, *IL-6* interleukin 6, *NOX-4* NADPH-oxidase 4, *OTC* ornithine carbamoyltransferase, mitochondria, *PRDX-2* peroxiredoxin-2, *TIMP-1* tissue inhibitor of metalloproteinases

In order to discriminate proteomic changes implicated in either IR-induced intestinal damage or rescuing through hPDSC administration, we performed comparative proteomic analysis using label-free quantification analysis with quadrupole time-of-flight (Q-TOF) mass spectrometry. As results, as seen in Fig. 5a, we pulled out whole proteomes from group 1 (*n* = 186), group 2 (*n* = 270), and group 3 (*n* = 238) and conducted a comparative gene ontology (GO) analysis. These 143 spots, including three groups in common were changed according to group condition (see Additional file 1: Table S3). Upon the serial comparative analysis, we categorized these proteome spots into five groups (Fig. 5b). Briefly, we could confirm that hPDSCs imposed anti-oxidative, cell differentiation, and tissue repair, whereas they resulted in the inhibition of signal transduction relevant to inflammation, oxidative stress, and ion transport and the changing patterns could be categorized into five patterns as seen in Fig. 5b, among which the following three pattern types were prominent, that is, proteome spots significantly decreased in group 2, whereas significantly increased in group 3 (Fig. 5c and Table 1), proteomic spots significantly increased in group 2, but significantly decreased in group 3 (Fig. 5d and Table 1), and proteomic spots were not changed in group 2, but significantly increased in group 3 (Fig. 5e and Table 1). The proteomic spots identified as proteomic biomarkers signifying “elevated following IR, but decreased following administration of hPDSCs” were phosphatidylethanolamine-binding protein 1 (PEBP1), apoptosis-associated speck-like protein containing CARD (PYCARD), glycerol 3-phosphate dehydrogenase [NAD (+)], cytoplasmic (GPD1) and ornithine carbamoyltransferase, mitochondria (OTC) (Fig. 5d and Table 1), and three proteomic spots, identified as proteomic biomarkers containing “no significant changes following IR, but increased following administration of hPDSCs” were tubulin-α-1B chain (TUBA1B), cytochrome *c* oxidase subunit 2 (MT-CO2), and peroxiredoxin-2 (PRDX-2) (Fig 5e and Table 1). To draw significant proteomic biomarkers with validation, proteomic biomarkers signifying “downregulated following IR, but increased following administration of hPDSCs”, IL-10, TIMP-1, and GST *mu* type 1 (GST *mu*1) were done with Western blot using homogenates from each group (Fig. 6a and b). As validation to these discoveries, as shown in Fig. 6a, the expression levels of IL-10 were decreased with irradiation (*P* < 0.001), but these levels were increased in group 3 (*P* < 0.05). When cells were irradiated, their destruction is enhanced through the activation of MMPs, especially MMP-2 or MMP-9, an enzyme pathway initiated by p53 [21]. TIMP-1 is a glycoprotein expressed from several tissues [22] as a natural inhibitor of MMPs [23], and has also been reported to reflect protection against IR-induced tissue damage. As shown in Fig. 6a, TIMP-1 was decreased in group 2 (*P* < 0.05), but its level increased following hPDSC administration (*P* < 0.0005). As already seen in Fig. 3c, MMP-2 activity was significantly increased in group 2. GST *mu* type—known as a strong anti-oxidant marker—was upregulated in group 3 whereas a decrease in expression of this protein was observed in group 2 (*P* < 0.05, Fig. 6b). Cancellation of NOX-4, OTC and GPD1 was validated as a biomarker predicting favorable response with hPDSC administration (Fig. 6c). As a biomarker unchanged as a result of IR treatment, but increased following hPDSC administration, GST (pi) and PRDX-2—known as a strongly anti-oxidative factor—was validated (*P* < 0.05, Fig. 6d).

**Table 1:** Label-free quantification analysis: novel biomarker candidates

Proteomes elevated with irradiation (G2) compared to control (G1), but decreased with hPDSC (G3)			
Protein description	G1	G2	G3
Fatty acid-binding protein, adipocyte	1	1.6125	0.7874
Heat shock protein 75 kDa, mitochondrial	1	1.7864	0.9751
RIKEN cDNA 2210010C04, isoform CRA_b	1	2.9038	1.1034
Neurobeachin-like protein 2	1	7.2087	3.2829
Gelsolin	1	9.6935	3.3595
Nucleolin	1	11.1004	5.0287
Phosphatidylethanolamine-binding protein 1	1	12.4758	1.8796
Protein 6530409C15Rik	1	15.1723	9.6556
Apoptosis-associated speck-like protein containing a CARD	1	24.5311	0.9818
Nucleoside diphosphate kinase B	1	25.2488	15.8437
Glycerol-3-phosphate dehydrogenase [NAD(+)], cytoplasmic	1	38.2966	21.2440
Tropomyosin alpha-3 chain	1	40.7211	0.30890
60S ribosomal protein L22	1	598.3902	127.068
Ornithine carbamoyltransferase, mitochondrial	1	249659	163892
Histone H4	1	609717	304923
Proteomes decreased after irradiation (G2) compared to control (G1), but increased with hPDSC (G3)			
Protein Mptx 2	1	0.1956	0.5902
Glutathione S-transferase Mu 1	1	0.2547	7.3523
Proteomes not changed with irradiation (G2) compared to control (G1), but increased with hPDSC (G3)			
Profilin-1	1	0.7039	3.2086
Myosin-10	1	0.8149	11.362
Tubulin alpha-1B chain	1	0.8376	17.0778
Macoilin	1	0.8532	6.89827
Cytochrome c oxidase subunit 2	1	0.9668	2.58191
Gastrotropin	1	1.09828	1.81219
Peptidyl-prolyl cis-trans isomerase A	1	1.3439	21.883
Maltase-glucoamylase	1	1.3488	6.0850
Peroxiredoxin-2	1	1.4326	4.2072
Histone H2A type 1-F	1	1.4423	4.6122
Calnexin	1	1.4684	2.8897

## Discussion

In contrast to previous reports that MSCs afforded the protective efficacies of MSCs against radiation enteropathy [17, 24–31], our results clearly showed that hPDSCs can be applied for either prophylactic or therapeutic purposes against radiation enteropathy. Also, we successfully elucidated potential biomarkers predicting a favorable response to stem cell therapy. Detailed summarization showed that hPDSCs, (i) significantly rescued mice from radiation syndrome by relieving systemic inflammatory response syndrome, (ii) significantly reduced the inflammation and apoptosis of cells within the small intestine subsequent to IR-induced robust oxidative stress, (iii) significantly afforded the recovery of the small intestine via either ameliorated apoptosis or enhanced anti-apoptosis, (iv) significantly resisted the loss of IR-induced stem cells essential for regeneration, and (v) cell therapy can be tailored through discovery of biomarkers predicting of favorable responses with hPDSCs. Similar to previous publications seeking solutions for radiation-induced intestinal toxicity through cell therapy, hPDSCs in the current study effectively enhanced or maintained the re-epithelization process along with significantly concerted actions of anti-inflammation, anti-oxidation, and anti-apoptosis.

Though beneficial effects have been reported with small molecules acting as either a prolyl hydroxylase inhibitor or a Rho kinase inhibitor (Y-27632) [26], autophage induction [31], natural products like hyaluronic acid, drugs like sucralfate [28], polaprezinc, coniferyl aldehyde as heat shock factor-1-inducing agent [29], amifostine [32], and tocotrienol [33], oral budesonide, *lactobacillus* probiotics [30] against radiation intestinal damage, substantial differences exist with our current study, that is, supplementing the loss of stem cells and concerted mechanisms for regeneration via stem cells might be fundamental and essential in radiation enteritis. Considering the unmet medical needs that current management of radiation-induced intestinal damage is only supportive and supplementary [9], intestinal SCs and bone marrow are very vulnerable to IR, and Lgr5^+^ stem cells are indispensable for radiation-induced intestinal regeneration [18], MSCs can play an important role in repairing tissue injury based on their self-renewal and multi-differentiation potential. Conclusively, current documentation of radiobiological effects of hPDSCs remains a challenge when seeking a safe strategy to either prevent or treat troublesome radiation enteropathy.

Furthermore, hPDSCs afforded significant protection from fatal acute radiation syndrome (Fig. 4f and g) beyond significant rescuing efficacy from IR-induced intestinal damage. Hence, hPDSCs protected irradiated mice by either inducing hematopoiesis or reducing apoptosis in affected organs. In this protection, three major mechanisms of MSC-induced hematopoiesis contributed, one is comparatively scarce cytokine-like granulocyte-macrophage colony-stimulating factor (GM-CSF), stromal cell-derived factor-1 (SDF-1), and IL-6 after irradiation, but make up for cytokine shortage by MSCs [34], the second is the recovery of the hematopoietic microenvironment destroyed by irradiation [35], and lastly, the regeneration of radiation-damaged bone marrow (BM) niche along with the protection of hematopoietic cells from apoptosis [25]. The classic understanding of radiation enteropathy was based on “the single target cell” concept around the 1920s and 1930s, but nowadays, extended to intestinal microvasculature, immune mechanisms, neuroimmune interactions, gut microbiome, the composition of the intraluminal contents, and other host factors [36]. Radiation predominantly kills rapidly proliferating cells such as progenitor cells in the intestinal crypts, leading to insufficient replacement of the villus epithelium [37] as seen in Fig. 2a, and induces many changes in endothelial cells including apoptosis, detached basement membrane, increased permeability, and interstitial fibrin deposition [38]. In this situation, SCs migrated to irradiation-exposed sites [39], produced vital growth factors and anti-oxidants in a paracrine manner [40], transdifferentiated to form damaged intestinal functional cells [41], and endowed authentic proliferative activities, which can open up the hope of exciting new cell therapeutic strategies as seen in Fig. 1d-f.

Various types of MSCs have been reported to be effective against radiation enteropathy including human adipose-derived MSCs [17], BM-derived MSCs (BMSCs) [42, 43], mesenchymal stromal cells [27, 44], placental stromal cells [25]. Horton et al. [45] showed that BMSCs could alter the progression of radiation-induced fibrosis by altering macrophage phenotype and suppressing local inflammation. As described by Auletta et al. [46], stem cells afforded the multi-potent and multi-therapeutic effects for host defense and MSCs homed significantly to injured sites to signal local cells to mitigate inflammation and preserve innate organ function. As seen in our models, local irradiation not only induces homing of human MSC at exposed sites, but also promotes their widespread engraftment to multiple organs, strongly supporting the use of MSCs to repair damaged normal tissues either following accidental irradiation or radiotherapy [39]. Though the signaling pathway responsible for these homing effects still remains unknown, it is inferred that molecular mediators such as chemokines and cytokines might orchestrate this process [47].

Though translational studies will be required to define the future clinical applications, from our study, we recommend thrice administration of intravenous 1 × 10^6^ hPDSCs every week. Though we have tried other kinds of MSCs, adipose-derived MSC, umbilical cord-derived MSG, and BM-derived MSC under the same experimental models, the conclusion was that current hPDSCs showed the best efficacy among these MSCs and showed the lowest immunogenicity compared to other kinds of allogenic MSCs (data not shown). Stem cell niche include both WNT and BMP signaling pathway, and the balance between their signaling is important within the intestine [48]. The WNT signaling pathway has emerged as a potential regulator of self-renewal for intestinal stem cells [49]. In the current study, we identified markers of the stem cell niche including Noggin, DLL4 and WNTs. As a result, the expression of Noggin was found to be remarkably increased in the hPDSC-treated group as compared to both the irradiated and control group and WNT6 slightly increased in the hPDSC-treated group (data was not shown).

Another important tip for future clinical application of hPDSCs came from our effort to discover biomarkers to predict the favorable response after hPDSC administration (Figs. 5 and 6), of which a validation study showed an increase in mucosal expression of IL-10, OTC1, mitochondrial sn-glycerol 3-phosphate dehydrogenase (mGPDH), TIMP-1, GSTM1, NOX4, and PRDX2 can be biomarkers significantly suggestive of favorable responses to hPDSCs. Looking at the biological action of discovered biomarkers, core mechanisms of hPDSCs against radiation enteropathy included efficient anti-inflammation and anti-oxidation and regeneration. Inferring more, IL-10, beyond being a well-known anti-inflammatory cytokine, is considered a key regulator of licensing regulatory T cells relevant to allogeneic stem cells [50] and stem cell renewal to promote their regeneration [51]; changes of OTC1 (though this gene is involved in neuropathology due to deficiency [52]), is only identified in one report relevant to roentgen irradiation [53], mGPDH1 as a ubiquinone-linked enzyme in the mitochondrial inner membrane (though the physiological role of this enzyme remains poorly defined in many tissues), might play a compensatory role in pathways for cytosolic regeneration of NAD^+^ and mechanisms for glycerol phosphate metabolism relevant to irradiation [54], and TIMP1, a tissue inhibitor of MMPs contributes to MSCs-induced healing [55]. In a recent publication by Haubner et al. [56], MSCs like ADSCs stimulated wound healing via increased TIMP1 and TIMPs in radiotherapy-associated impaired wound healing. GSTM1 were already reported to be genetic predictors of long-term toxicities after radiation therapy [57], NOX-4 as inducer of total body irradiation-induced hematopoietic genomic instability [58], and PRDX2 as UV responses and radiation in human skin [59].

## Conclusions

Conclusively, hPDSCs, featured with relative low immunogenicity compared to other kinds of allogenic MSCs, afforded prophylactic and therapeutic actions against troublesome radiation enteropathy through well-orchestrated actions including anti-inflammation, anti-oxidation, cytoprotection, and regeneration. Though further detailed clinical trials are required, our identification of biomarkers predicting favorable responses can add to the tailored application of SC therapeutics in alleviating radiation enteropathy.

## Additional file

Additional file 1: Table S1. Primers used in the current study. **Table S2.** Criteria used for pathological grading in the current study. **Table S3.** Analysis of proteomics; proteome showing difference between G2 and G3 (*P* < 0.05). (DOC 185 kb)

## Abbreviations

Bcl-2: B-cell lymphoma 2
BMSCs: Bone marrow-derived stem cells
CD31: Cluster of differentiation 31
COX-2: Cyclooxygenase-2
EGFR: Epidermal growth factor receptor
GI: Gastrointestinal
GO: Gene ontology
GPD1: Glycerol 3-phosphate dehydrogenase [NAD (+)], cytoplasmic
GST *mu*: Glutatione-S-transferase mu
GVHD: Graft-versus-host disease
hPDSCs: Human placenta-derived mesenchymal stem cells
IFN-γ: Interferon gamma
IκBα: Inhibitor of kappa B
IL-10: Interleukin-10
IL-6: Interleukin 6
iNOS: Nitric oxide synthase
IR: Ionizing radiation
mGPDH: Mitochondrial sn-glycerol 3-phosphate dehydrogenase
MMP-2: Matrix metalloproteinase-2
MT-CO2: Cytochrome c oxidase subunit 2
NF-κB: Nuclear factor-κB
NOX-4: NADPH-oxidase 4
OTC: Ornithine carbamoyltransferase, mitochondria
PARP: Poly (ADP-ribose) polymerase
PEBP1: Phosphatidylethanolamine-binding protein 1
PRDX-2: Peroxiredoxin-2
PYCARD: Apoptosis-associated speck-like protein containing CARD
Q-TOF: Quadrupole time-of-flight
SCs: Stem cells
STAT3: Signal transducer and activator of transcription 3
TIMP-1: Tissue inhibitor of metalloproteinases
TNF-α: Tumor necrosis factor alpha
TUBA1B: Tubulin-α-1B chain
TUNEL: Terminal deoxynucleotidyl transferase-mediated dUTP nick-end labeling
