# Supplementary material for: The efficacy of human placenta-derived mesenchymal stem cells on radiation enteropathy along with proteomic biomarkers predicting a favorable response
Source: Stem Cell Res Ther. 2017 May 2;8:105. doi: 10.1186/s13287-017-0559-5 (PMC5414323; doi:10.1186/s13287-017-0559-5)
Supplement: Additional file 1: Table S1. — Primers used in the current study. Table S2. Criteria used for pathological grading in the current study. Table S3. Analysis of proteomics; proteome showing difference between G2 and G3 (P < 0.05). (DOC 185 kb) [file 13287_2017_559_MOESM1_ESM.doc]

*Additional file information*

**The efficacy of human placenta-derived mesenchymal stem cells on radiation enteropathy along with proteomic biomarkers predicting a favorable response**

**Short Title: Therapeutic effect of MSC on radiation enteropathy**

[Keywords: Placenta-derived mesenchymal stem cells, Radiation enteropathy, Regeneration, Biomarkers]

Young-Min Han^1^, Jong-Min Park^1^, Yong Soo Choi^2^, Hee Jin^3^, Yun-Sil Lee^3^, Na-Young Han^4^, Hookeun Lee^4^ and Ki Baik Hahm^1, 5^

^1^CHA Cancer Prevention Research Center, CHA Bio Complex, Seongnam, Korea

**^2^**Department of Applied Bioscience, CHA University, Seongnam, Korea

**^3^** Graduated School of Pharmaceutical Sciences, Ewha Womans University, Seoul, Korea

^4^ Lee Gil Ya Cancer and Diabetes Institute, College of Pharmacy, Gachon University, Incheon, Korea

^5^Digestive Disease Center, CHA Bundang Medical Center, CHA University, Seongnam, Korea

**Correspondence to: Professor Ki Baik Hahm, MD. Ph.D**

CHA Cancer Prevention Research Center, CHA University CHA Bio Complex,

335 Pangyo-ro, Bundang-ku, Seongnam, Kyunggi-do, Korea 463-712

Tel.: +82 31 881 7251, Fax: +82 31 881 7250

E-mail: hahmkb@cha.ac.kr

**Additional file 1: Table S1. Primers used in the current study**

| Gene name | Sequence |
| --- | --- |
| *GAPDH* | Forward 5'- AAT GTA TCC GTT GTG GAT CT -3' |
|  | Reverse 5'- TCC ACC ACC CTG TTG CTG TA -3' |
| *IL-6 (Q)* | Forward 5'- CCACTTCACAAGTCGGAGGCTTA -3' |
|  | Reverse 5'- GCAAGTGCATCATCGTTGTTCATAC -3' |
| *IL-10* | Forward 5'- CCA GTT TTA CCT GGT AGA AG -3' |
|  | Reverse 5'- AGG TCC TGG AGT CCA GAC TC -3' |
| *TNF-α (Q)* | Forward 5'- ATG AGC ACA GAA AGC ATG ATC -3' |
|  | Reverse 5' - TAC AGG CTT GTC ACT CGA ATT -3' |
| *IFN-γ* | Forward 5'- CTC TGA GAC AAT GAA CGC T -3' |
|  | Reverse 5'- AAA GAG ATA ATC TGG CTC TGC -3' |
| *IFN-γ (Q)* | Forward 5'- CGGCACAGTCATTGAAAGCCTA -3' |
|  | Reverse 5'- GTTGCTGATGGCCTGATTGTC -3' |
| *COX-2* | Forward 5'- CAT CCT GCC AGC TCC ACC GC -3' |
|  | Reverse 5'- GGG AGG AAG GGC CCT GGT GT -3' |
| *iNOS* | Forward 5'- GTG GTG ACA AGC ACA TTT GG -3' |
|  | Reverse 5'- GGC TGG ACT TTT CAC TCT GC -3' |

Q denoted primer for qRT-PCR

**Additional file 1: Table S2. Criteria used for pathological grading in the current study**

| **Score**  **Index** | **0** | **1** | **2** | **3** | **4** |
| --- | --- | --- | --- | --- | --- |
| **Ulceration** | 0 | 1/4 | 1/2 | 1 | Perforation |
| **Villi loss** | 0 | < 30 % | 30 ~ 50 % | 50 ~ 70 % | 70 % < |
| **Inflammation** | 0 | < 30 % | 30 ~ 50 % | 50 ~ 70 % | 70 % < |

**Additional file 1: Table S3. Analysis of proteomics; proteome showing difference between G2 and G3 (*P* < 0.05)**

| Protein description | G1 | G2 | G3 |
| --- | --- | --- | --- |
| Protein Mptx2 | 1 | 0.1956 | 0.5902 |
| Glutathione S-transferase Mu 1 | 1 | 0.2548 | 7.3523 |
| Ornithine aminotransferase, mitochondrial | 1 | 0.6103 | 0.5761 |
| Hemoglobin alpha, adult chain 1 | 1 | 0.6130 | 0.5361 |
| Profilin-1 | 1 | 0.7039 | 3.2086 |
| Myosin-10 | 1 | 0.8149 | 11.3629 |
| Tubulin alpha-1B chain | 1 | 0.8376 | 17.0778 |
| Macoilin | 1 | 0.8533 | 6.8983 |
| Cytochrome c oxidase subunit 2 | 1 | 0.9669 | 2.5819 |
| Gastrotropin | 1 | 1.0983 | 1.8122 |
| Peptidyl-prolyl cis-trans isomerase A | 1 | 1.3439 | 21.8834 |
| Maltase-glucoamylase | 1 | 1.3489 | 6.0850 |
| Peroxiredoxin-2 | 1 | 1.4327 | 4.2072 |
| Histone H2A type 1-F | 1 | 1.4424 | 4.6122 |
| Calnexin | 1 | 1.4685 | 2.8898 |
| Annexin A4 | 1 | 0.6938 | 0.6811 |
| Creatine kinase U-type, mitochondrial | 1 | 0.7396 | 0.6540 |
| Citrate synthase, mitochondrial | 1 | 0.7768 | 0.6269 |
| Annexin A2 | 1 | 0.9720 | 1.1354 |
| Heat shock protein HSP 90-alpha | 1 | 1.1166 | 0.8315 |
| D-beta-hydroxybutyrate dehydrogenase, mitochondrial | 1 | 1.1616 | 0.7900 |
| Heterogeneous nuclear ribonucleoprotein K | 1 | 1.1790 | 1.0996 |
| Platelet-derived growth factor subunit B | 1 | 1.2733 | 1.6582 |
| Heterogeneous nuclear ribonucleoprotein A3 | 1 | 1.3207 | 1.0181 |
| Malate dehydrogenase, mitochondrial | 1 | 1.3530 | 1.6575 |
| Alpha-actinin-1 | 1 | 1.4246 | 1.4768 |
| Protein disulfide-isomerase A3 | 1 | 1.4873 | 1.9623 |
| Fatty acid-binding protein, adipocyte | 1 | 1.6125 | 0.7874 |
| Heat shock protein 75 kDa, mitochondrial | 1 | 1.7865 | 0.9751 |
| RIKEN cDNA 2210010C04, isoform CRA_b | 1 | 2.9039 | 1.1040 |
| Neurobeachin-like protein 2 | 1 | 7.2088 | 3.2829 |
| Gelsolin | 1 | 9.6936 | 3.3595 |
| Nucleolin | 1 | 11.1005 | 5.0288 |
| Phosphatidylethanolamine-binding protein 1 | 1 | 12.4758 | 1.8796 |
| Protein 6530409C15Rik | 1 | 15.1724 | 9.6557 |
| Apoptosis-associated speck-like protein containing a CARD | 1 | 24.5312 | 0.9819 |
| Nucleoside diphosphate kinase B | 1 | 25.2489 | 15.8437 |
| Glycerol-3-phosphate dehydrogenase [NAD(+)], cytoplasmic | 1 | 38.2967 | 21.2440 |
| Tropomyosin alpha-3 chain | 1 | 40.7212 | 0.3089 |
| 60S ribosomal protein L22 | 1 | 598.3902 | 127.0684 |
| Ornithine carbamoyltransferase, mitochondrial | 1 | 249659 | 163892 |
| Histone H4 | 1 | 609717 | 304923 |
| Keratin, type II cytoskeletal 8 | 1 | 1.5517 | 2.2454 |
| Creatine kinase B-type | 1 | 1.6438 | 1.3993 |
| Cytosolic non-specific dipeptidase | 1 | 1.6993 | 1.7369 |
| Aminopeptidase N | 1 | 1.8260 | 1.9691 |
| ADP/ATP translocase 2 | 1 | 1.9037 | 1.6579 |
| Collagen alpha-1(I) chain | 1 | 1.9324 | 2.6541 |
| ATP synthase subunit beta, mitochondrial | 1 | 1.9962 | 2.2665 |
| ADP/ATP translocase 1 | 1 | 2.0516 | 2.1409 |
| Microsomal triglyceride transfer protein large subunit | 1 | 2.0594 | 2.6486 |
| Tubulin alpha-1A chain | 1 | 2.0609 | 1.6827 |
| Myosin-11 | 1 | 2.0873 | 3.0739 |
| Hemoglobin subunit beta-1 | 1 | 2.1595 | 1.6675 |
| Transketolase | 1 | 2.1749 | 1.8099 |
| 14-3-3 protein zeta/delta | 1 | 2.2320 | 2.6129 |
| Elongation factor 1-alpha 1 | 1 | 2.2745 | 1.9505 |
| 78 kDa glucose-regulated protein | 1 | 2.3571 | 2.0880 |
| Protein disulfide-isomerase | 1 | 2.3728 | 2.8584 |
| Phosphoglycerate kinase 1 | 1 | 2.4018 | 2.0666 |
| Malate dehydrogenase, cytoplasmic | 1 | 2.4178 | 3.1328 |
| ATP synthase subunit alpha, mitochondrial | 1 | 2.4706 | 2.6903 |
| Aldehyde dehydrogenase X, mitochondrial | 1 | 2.7976 | 3.9692 |
| Fatty acid-binding protein, liver | 1 | 2.8719 | 2.9255 |
| Elongation factor 2 | 1 | 2.9859 | 3.2301 |
| 14-3-3 protein beta/alpha | 1 | 2.9914 | 2.5467 |
| Filamin-A | 1 | 3.0032 | 4.4011 |
| Glyceraldehyde-3-phosphate dehydrogenase | 1 | 3.3079 | 2.7368 |
| Galectin-2 | 1 | 3.4863 | 3.5248 |
| Alpha-enolase | 1 | 3.6723 | 4.7293 |
| Phosphoglycerate mutase 1 | 1 | 3.7814 | 3.5322 |
| Actin, aortic smooth muscle | 1 | 3.9917 | 4.9536 |
| Galectin-4 | 1 | 4.2899 | 4.7625 |
| Keratin, type I cytoskeletal 20 | 1 | 4.4041 | 3.7380 |
| Pyruvate kinase PKM | 1 | 4.4740 | 5.0029 |
| Olfactory receptor | 1 | 4.9070 | 3.7475 |
| 40S ribosomal protein S19 | 1 | 4.9965 | 4.0182 |
| Ubiquitin-60S ribosomal protein L40 | 1 | 5.2456 | 4.0973 |
| Calreticulin | 1 | 5.2524 | 6.7149 |
| Retinol-binding protein 2 | 1 | 5.2796 | 4.3788 |
| Endoplasmin | 1 | 5.3549 | 5.0003 |
| Peroxiredoxin-1 | 1 | 5.4576 | 4.9258 |
| L-lactate dehydrogenase A chain | 1 | 5.6290 | 5.1774 |
| Hemoglobin subunit alpha | 1 | 5.6387 | 5.8195 |
| Hemoglobin subunit zeta | 1 | 5.7791 | 5.9366 |
| Ezrin | 1 | 5.8340 | 6.6015 |
| Fructose-bisphosphate aldolase A | 1 | 5.8896 | 6.0358 |
| Heat shock cognate 71 kDa protein | 1 | 6.2173 | 5.7774 |
| Galectin-6 | 1 | 6.3751 | 9.1948 |
| Diacylglycerol kinase | 1 | 6.4766 | 9.3292 |
| Superoxide dismutase [Cu-Zn] | 1 | 6.6026 | 5.6115 |
| 60S acidic ribosomal protein P2 | 1 | 6.6485 | 7.6050 |
| Alpha-amylase 1 | 1 | 6.6588 | 4.8621 |
| Alpha-actinin-4 | 1 | 6.7176 | 6.0445 |
| 60S ribosomal protein L11 | 1 | 6.9593 | 5.9060 |
| Nucleoside diphosphate kinase A | 1 | 7.3621 | 7.0429 |
| Cytochrome b-c1 complex subunit 2, mitochondrial | 1 | 7.6910 | 8.2292 |
| Glucose-6-phosphate isomerase | 1 | 7.9481 | 6.1822 |
| Serum albumin | 1 | 8.0337 | 6.0795 |
| Aconitate hydratase, mitochondrial | 1 | 8.6330 | 8.1877 |
| Eukaryotic initiation factor 4A-I | 1 | 8.9304 | 10.4439 |
| Alpha-internexin | 1 | 8.9741 | 9.4738 |
| Aldehyde dehydrogenase, mitochondrial | 1 | 9.0757 | 10.4857 |
| Fructose-bisphosphate aldolase B | 1 | 9.3399 | 8.6453 |
| Keratin, type II cytoskeletal 2 epidermal | 1 | 10.1817 | 14.6953 |
| Heterogeneous nuclear ribonucleoproteins A2/B1 | 1 | 10.6675 | 9.1236 |
| Cytochrome c1, heme protein, mitochondrial | 1 | 10.6770 | 9.2104 |
| Amine oxidase [flavin-containing] A | 1 | 11.0154 | 10.5899 |
| Vimentin | 1 | 12.6121 | 10.8679 |
| Protein Fcg | 1 | 14.4195 | 17.6302 |
| Collagen alpha 3 chain type VI | 1 | 14.8021 | 15.4984 |
| GDP-mannose 4,6 dehydratase | 1 | 14.9693 | 17.9892 |
| ATP synthase subunit O, mitochondrial | 1 | 15.0802 | 10.5414 |
| Heat shock-related 70 kDa protein 2 | 1 | 15.9216 | 13.5397 |
| 40S ribosomal protein S3 | 1 | 16.0717 | 11.5014 |
| UDP-glucuronosyltransferase 1-7C | 1 | 18.2075 | 27.1012 |
| Tropomyosin 1 | 1 | 18.5836 | 22.8007 |
| Cytosol aminopeptidase | 1 | 21.5242 | 15.2927 |
| Cytochrome b5 | 1 | 24.1768 | 29.5156 |
| Cadherin-17 | 1 | 24.3214 | 24.3478 |
| Destrin | 1 | 25.4449 | 22.8272 |
| ATP synthase subunit gamma, mitochondrial | 1 | 26.0123 | 19.0930 |
| Myosin light polypeptide 6 | 1 | 28.7607 | 39.4045 |
| Potassium-transporting ATPase alpha chain 2 | 1 | 29.7900 | 23.0891 |
| Tropomyosin 2, beta, isoform CRA_b | 1 | 39.6433 | 51.5175 |
| Myosin regulatory light polypeptide 9 | 1 | 47.5017 | 44.2782 |
| Tubulin beta-3 chain | 1 | 58.1838 | 51.0615 |
| Tubulin beta-1 chain | 1 | 285274 | 235908 |
| Glutamate dehydrogenase 1, mitochondrial | 1 | 735348 | 690517 |
| Tubulin beta-2A chain | 1 | 1.5559 | 3.6160 |
| Alpha-defensin 5 | 1 | 1.8343 | 48.3678 |
| Myosin regulatory light chain 12B | 1 | 1.9543 | 5.0917 |
| 40S ribosomal protein S7 | 1 | 2.2893 | 10.0183 |
| 10 kDa heat shock protein, mitochondrial | 1 | 2.3755 | 11.5069 |
| 14-3-3 protein epsilon | 1 | 3.0880 | 23.1423 |
| Fatty acid-binding protein, intestinal | 1 | 3.5677 | 6.3611 |
| Histone H2B type 1-B | 1 | 4.2189 | 8.6242 |
| Villin-1 | 1 | 4.6807 | 10.7515 |
| Triosephosphate isomerase | 1 | 4.8024 | 8.3947 |
| Desmin | 1 | 4.9142 | 10.2864 |
| Spermatogenesis-associated protein 7 homolog | 1 | 6.2441 | 23.9839 |
| Tropomyosin beta chain | 1 | 6.5339 | 58.4243 |
| Na(+)/H(+) exchange regulatory cofactor NHE-RF1 | 1 | 7.0356 | 15.3701 |
| Zymogen granule membrane protein 16 | 1 | 7.6540 | 22.9257 |
| Glyceraldehyde-3-phosphate dehydrogenase, testis-specific | 1 | 7.8658 | 49.7071 |
| Myosin light chain 6B | 1 | 7.9318 | 346 |
| Transgelin | 1 | 8.2439 | 14.4641 |
| Myosin-9 | 1 | 11.2131 | 18.2446 |
| Stress-70 protein, mitochondrial | 1 | 12.4534 | 28.5856 |
| ADP-ribosylation factor 1 | 1 | 13.4822 | 46.6719 |
| Phosphate carrier protein, mitochondrial | 1 | 14.6714 | 31.3576 |
| Keratin, type I cytoskeletal 13 | 1 | 16.0624 | 107.7093 |
| Mitochondrial amidoxime reducing component 2 | 1 | 17.2997 | 47.8450 |
| Proteasome-associated protein ECM29 homolog | 1 | 26.9432 | 60.9485 |
| Rab GDP dissociation inhibitor beta | 1 | 31.1461 | 262 |
